# Adaptation and Therapeutic Exploitation of the Plasma Membrane of African Trypanosomes

**DOI:** 10.3390/genes9070368

**Published:** 2018-07-20

**Authors:** Juan F. Quintana, Ricardo Canavate Del Pino, Kayo Yamada, Ning Zhang, Mark C. Field

**Affiliations:** School of Life Sciences, University of Dundee, Dow Street, Dundee, DD1 5EH, UK; j.f.z.quintanaalcala@dundee.ac.uk (J.F.Q.); r.canavatedelpino@dundee.ac.uk (R.C.d.P.); k.ono@dundee.ac.uk (K.Y.); n.v.zhang@dundee.ac.uk (N.Z.)

**Keywords:** *Trypanosoma brucei*, surface proteome, endomembrane system, endocytosis, drug development, nanobodies

## Abstract

African trypanosomes are highly divergent from their metazoan hosts, and as part of adaptation to a parasitic life style have developed a unique endomembrane system. The key virulence mechanism of many pathogens is successful immune evasion, to enable survival within a host, a feature that requires both genetic events and membrane transport mechanisms in African trypanosomes. Intracellular trafficking not only plays a role in immune evasion, but also in homeostasis of intracellular and extracellular compartments and interactions with the environment. Significantly, historical and recent work has unraveled some of the connections between these processes and highlighted how immune evasion mechanisms that are associated with adaptations to membrane trafficking may have, paradoxically, provided specific sensitivity to drugs. Here, we explore these advances in understanding the membrane composition of the trypanosome plasma membrane and organelles and provide a perspective for how transport could be exploited for therapeutic purposes.

## 1. Introduction

**Figure 1:**
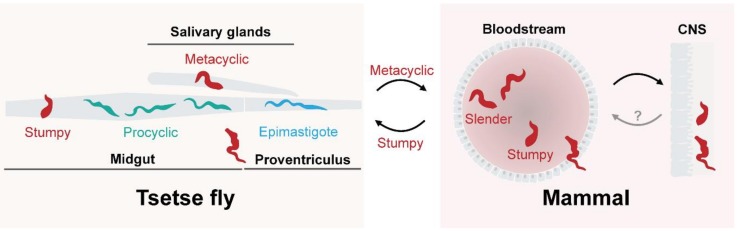
Simplified life cycle of *Trypanosoma brucei*. Parasites in the stumpy stage differentiate into procyclic forms inside the midgut of the tsetse fly. Procyclic forms of the parasite express a procyclin surface that changes throughout the infection of the midgut. On route to the salivary glands, trypanosomes cross the proventriculus, differentiate into epimastigotes, and switch the procyclin coat to bloodstream stage alanine-rich protein BARP. The final stage of infection in the tsetse fly takes places in the salivary glands; the parasites differentiate into metacyclic forms and express variant surface glycoproteins (VSGs), ready to infect a new host. Through a blood meal, trypanosomes reach the bloodstream of a mammalian host and differentiate into the replicative, long and slender form. This may also involve the skin, lymphatics and adipose tissues, but which are omitted for simplicity. As the levels of parasitemia increase, trypanosomes differentiate into the infective, non-replicative short and stumpy form. Trypanosomes are also able to reach the cerebrospinal fluid and to cross the blood brain barrier into the central nervous system (CNS). It is unclear if this population is in equilibrium with the bloodstream stages. The dominant proteins in each stage of the infection are indicated by colour: VSG; red, BARP; blue and procyclin; teal.

Over one billion years ago, a group of unicellular organisms emerged and ultimately arose as trypanosomatids, featuring a singular flagellum and a kinetoplastid [1]. Their position as a highly divergent taxon in eukaryotic evolution provides unique opportunities for studying the mechanisms of evolution of many aspects of the eukaryotic cell [2,3]. Moreover, being almost exclusively parasitic, trypanosomes impose a significant burden on public health and economic advancement. The African trypanosomes are the causative agents of Human African trypanosomiasis (HAT), or “sleeping sickness”, and animal African trypanosomiasis (AAT), or “Nagana”, and are transmitted by the tsetse fly [4,5] (Figure 1). 

These extracellular parasites have evolved multiple mechanisms to successfully establish and maintain an infection in both insect and mammal hosts [6]. The most sophisticated of these is the frequent remodelling of the surface proteome during the course of infection in mammalian hosts coupled with the removal of surface-bound immune effectors [4]. During the transition through the vertebrate host, the plasma membrane of *Trypanosoma brucei* spp. is characterized by the presence of a dense coat that is composed mostly of variant surface glycoproteins (VSGs) [4]. VSGs are periodically switched allowing for the parasite to present distinct surface epitopes at any given time, conferring a competitive advantage over the immune system [4]. Moreover, the VSG coat provides a physical barrier between immune effector complexes and non-varying surface proteins, such as nutrient and ion transporters and receptors [4,5]. Hence, trypanosomes shield themselves from their environment and present just a single invagination at the plasma membrane, the flagellar pocket (FP), to provide the sole point of active exchange with the cell interior [5]. 

**Figure 2:**
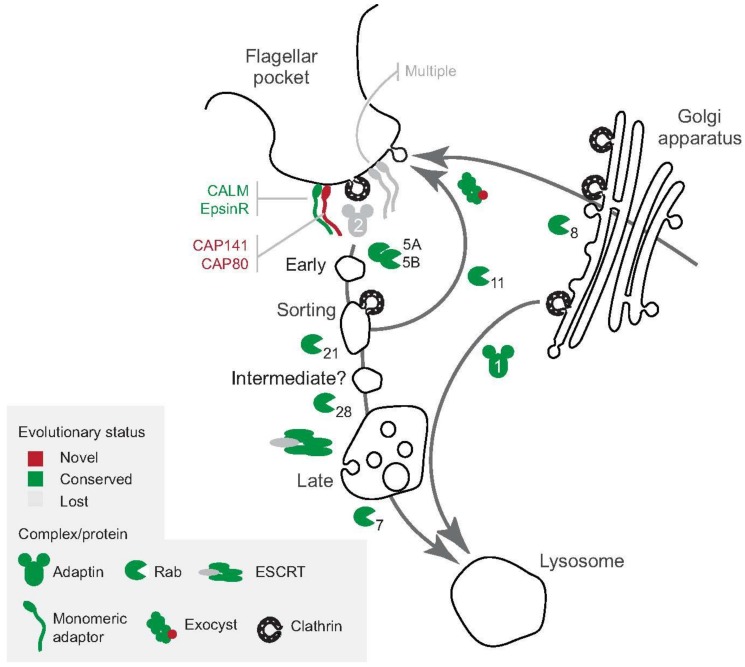
Organization of the endomembrane system of bloodstream form of *T. brucei* sp. A simplified schematic representation of the trypanosome endomembrane system is shown, with the flagellar pocket at the top of the panel, and including other intracellular organelles, such as the trans-Golgi network (TGN), early, sorting and intermediate endosomes, and the lysosome. Intracellular trafficking routes are indicated by arrows, with the arrowheads pointing towards the destination of traffic. The endocytic route is depicted from the pocket to various intracellular organelles, whereas the exocytic route is depicted from intracellular organelles (TGN, endosomes) to the surface. The cohort of conserved trafficking-related protein between trypanosomes and higher eukaryotes is shown in green, whereas lineage-specific proteins, i.e. those present only in trypanosomes (and possibly a few additional taxa) are indicated in red. Similarly, those components not detected in trypanosomes, and therefore thought to be lost in these organisms (e.g. the adaptor protein 2 complex) are shown in grey. Rab proteins are also shown and positioned based on the step that they mediate as well as localisation data (e.g. Rab5 in early endosomes, Rab11 in sorting/recycling endosomes). Note that not all proteins and complexes shown are discussed in the text but are present for completeness. Abbreviations: ESCRT; endosomal sorting complexes required for transport, CALM; clathrin assembly lymphoid myeloid leukemia, CAP; clathrin-associated proteins, EpsinR; Eps15-interacting protein (Epsin)-related protein. Numbers in endosomal structures (early, sorting, intermediate, and late) represent the corresponding Rab protein (e.g. Rab5A, Rab5B, Rab11, Rab21, Rab28, etc.).

Remodeling of the trypanosome surface is supported by a highly efficient endocytic and exocytic apparatus and the underlying endomembrane system. Both the endocytic and exocytic systems of *T. brucei* possess canonical, highly conserved proteins, including clathrin and clathrin-interacting protein partners, adaptor proteins, tethering complexes, and Rab proteins, together with specialized lineage-specific components (Figure 2) [7,8,9]. Continuous and rapid endocytosis ensures that immune complexes that are formed on the cell surface are internalized and degraded rapidly in acidic intracellular compartments [7,8,9]. Alongside, the recycling system ensures that proteins, including VSGs, are swiftly returned to the surface to maintain the coat [8]. Additionally, the exocytic system must meet the requirement for de novo surface components, particularly relevant during cell growth and division, as well as VSG switching [8,10,11]. The concerted functions of both endocytosis and exocytosis are paramount to ensuring the fidelity of the cell surface proteome at any given time [7,8,9]. Understanding the mechanisms of surface proteome homeostasis in trypanosomatids has also become relevant for understanding those processes underpinning drug sensitivity and has the potential for exploitation for therapeutic purposes. For example, high endocytic capacity represents an opportunity for the efficient delivery of chemotherapeutic agents and may bypass potential treatment failure through the emergence of resistance [12]. 

Here, we present an integrated view of the surface proteome of *T. brucei*, with special emphasis on the plasma membrane and endomembrane interface. Similarly, we consider how the surface proteome, and its homeostatic maintenance, is key in providing effective mechanisms against recognition by the immune system during infection and describe the most recent findings providing a new depiction of how different trypanocidal agents function within trypanosomes.

## 2. A Dynamic Surface for Host and Environmental Interactions

The plasma membrane is the interface between the environment and the cell. The maintenance of the surface composition in trypanosomes, as in any eukaryotic cell, is only possible due to a large and highly specialized endomembrane system, broadly constituted by the endoplasmic reticulum (ER), the trans-Golgi network (TGN), and various endolysosomal organelles [5,8,13]. From a cellular point of view, the *T. brucei* ER is distributed throughout the cytoplasm, whereas multiple vesicular structures, including early, late, and recycling endosomes, alongside the TGN, are located in close proximity to the FP [5,8,13]. Similarly, the single lysosome is normally located close to the nucleus, with the result that the endomembrane system is highly polarized [5,8,13]. 

One of the key features of the *T. brucei* mammalian infective form is rapid and tightly controlled endocytic and recycling machineries (Figure 2). In trypanosomes, initial endocytic steps are exclusively mediated by clathrin and independent of adaptor protein 2 (AP-2), which associates with clathrin for the specific purpose of concentrating receptors within clathrin-coated pits in higher eukaryotes [14,15]. Clathrin-mediated endocytosis (CME) is the most evolutionarily ancient endocytic mechanism known, and in many lineages the sole mechanism for internalization [16]. Additionally, trypanosomes also possess a cohort of proteins that interact with clathrin to facilitate transport; some of which are conserved and others are trypanosomatid-specific [17]. Interestingly, the *T. brucei* genome does not encode orthologues for AP-2 subunits [17] and indicates that the initial steps of endocytosis are likely non-selective [17]. A conserved cohort characterized as bona fide interacting partners of clathrin includes adaptor protein 1 (AP-1) γ subunit (Tb917.4.760), AP-1 β subunit (Tb927.10.8040), AP-1 μ subunit (Tb927.7.3180), epsinR (Tb927.11.670), orthologues of endosomal SNAP (soluble NSF (N-ethylmaleimide Factor) Attachment protein) Receptor SNARE, Rab and ADP ribosylation factor(ARF) GTPase-activating protein (SMAP) (Tb927.11.9180), and the *T. brucei* AP-2 associated kinase-like (TbAAKL) (Tb927.11.9180) [17] (Figure 2). TbAAKL is particularly interesting as it is a pseudokinase in trypanosomes rather than an active AP-2 kinase, suggesting that there are additional roles for this protein beyond interaction with AP-2 [17]. By contrast, a second cohort of clathrin-interacting proteins have been described that are restricted to trypanosomes, several of which have clear roles in endocytosis and organization of the endomembrane system [17,18]. One key observation in this regard is that Rab5/Rab11-containining endosomes are important for sorting of VSGs and invariable surface glycoproteins (ISGs) during their internalization and recycling [19,20] (Figure 2).

Rab proteins are spatiotemporal regulators of membrane transport and have also served as valuable markers for endomembrane compartments in trypanosomes [21]. In the context of surface remodeling, Rab5 emerges as a key component during the early steps of endocytic trafficking [8,22,23]. *T. brucei* has two isoforms of Rab5, Rab5A (Tb927.10.12960) and Rab5B (Tb927.11.4570), which co-localize to the same structures in procyclic form (PCF) but are in different endosomal compartments in bloodstream form (BSF) [7,24,25], where these two endosomal populations receive distinct cargoes. For example, Rab5A, but not Rab5B, endosomes contain VSG [25] (Figure 2). These findings support a model in which the sorting of endocytosed surface proteins is through different endosomal structures, a form of functional diversion, perhaps to allow for a continuous recycling of VSG without disturbing the adequate internalization of other surface proteins [25,26]. This is also supported by the presence of clathrin buds on endosomal structures that appear to exclude VSG, suggesting that the recognition of trans-membrane domain proteins may take place at internal compartments and possibly would be mediated by AP-1 [8,27,28]. Subsequently, the endocytic process involves transitioning through additional compartments that are defined by Rab21 (Tb927.10.1520), Rab28 (Tb927.6.3040), and finally Rab7 (Tb927.9.11000) [29,30,31]. Quite what the specific functions of these steps are remains to be elucidated but control does appear to be mediated by the vacuolar protein sorting 9 (Vps9) domain proteins, such as Alsin (Tb927.3.2430) and Rabex5 + GAPVD1 (Tb927.10.10020), a mechanism that is conserved with mammalian cells [32]. 

Another subset of Rab proteins, Rab11 (Tb927.8.4330) and Rab4 (Tb927.11.13750), are intimately involved in the recycling of proteins from early endosomes to the plasma membrane (PM) [33,34,35]. However, there is conflictive evidence regarding the role of Rab11 in the recycling of trans-membrane proteins and glycosylphosphatidylinositol (GPI)-anchor proteins [33,34,35], which clearly evidence the need to further characterize the role of Rab11 in recycling in a more detailed manner. Nevertheless, the crosstalk between the early, Rab5-containing early endosomes and the Rab11-containing recycling endosomes are thought to be mediated by both conserved proteins as well as potentially lineage-specific proteins, such as 5-azacytidine-induced-1 (TbAZI1) (Tb09.211.4830) and Rab11-binding protein of 74 kDa (RBP74) (Tb927.5.1640), respectively [34]. Importantly, several Rab11-interacting partners, including Sec15 (Tb11.02.4970), connect the Rab11-containing endosomes to the exocyst [11], a complex that is important in mediating the late steps of exocytosis [11] (Figure 2). 

## 3. The Major Surface Proteins and Their Trafficking Itinerates

**Figure 3:**
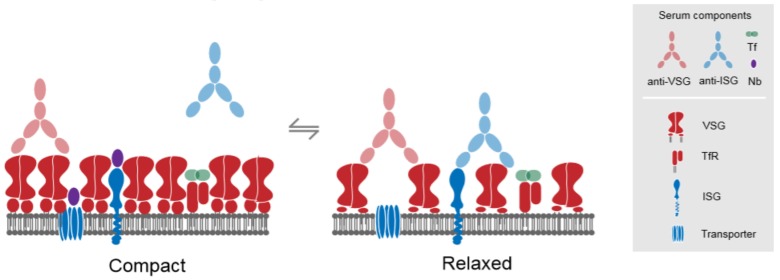
Arrangement of the VSG coat at the cell surface. VSGs form a physical barrier on the plasma membrane to protect the underlying and invariable membrane proteins, thereby preventing the exposure of potentially highly antigenic proteins. During VSG switching and other dynamic conditions, the surface must accommodate a greater number of VSG molecules, which is thought to be achieved by tightly packing VSG under a “compact” conformation. When the VSG switching program is completed and the old VSG is completely removed from the surface, the density of the newly formed VSG coat is reduced, leading to a “relaxed” conformation. It is likely that these conformations are dynamic at the steady-state level, and have important consequences for immune recognition, so that the antibody against invariant determinants (blue) is more likely in the relaxed state. Abbreviations: VSG, Variable Surface Glycoprotein; ISG, Invariant Surface Glycoproteins, Tf, Transferrin; TfR, Transferrin Receptor; Nb, Nanobodies.

VSGs are homodimeric ~50–55 kDa GPI-anchored proteins [4,5,36]. Each monomer is connected to the GPI-anchor by a flexible peptide linker [37], allowing the protein to exist in at least two conformational states; “compact”, characterized by tight protein packing, or “relaxed”, where the space between homodimers increases [37] (Figure 3). The flexibility and presence of at least two conformational states have implications for VSG movement at the surface. A recent study has shown that at least two populations can be measured, exhibiting differential lateral movement, one with slow dynamics and another population displaying faster movement, similar to that observed for lipids in the plasma membrane [37]. These results are consistent with a proposed model for structural flexibility of VSGs, and suggest that the “relaxed” and “compact” states diffuse in the plasma membrane at different rates [26,37]. 

VSGs are continually switched during the course of an infection [5,36]. This antigenic variation is the most sophisticated mechanism by which trypanosomatids evade being recognized by the host immune system [5,36]. Antigenic variation is achieved by means of a hypervariable N-terminal domain, which faces the extracellular space, when compared to the more conserved C-terminal domain, located under the dense VSG coat [5,36]. The process of antigenic switching implies that trypanosomes must survive with at least two different VSGs co-existing at the PM during the transition from one VSG to another [4,38,39]. It is plausible that the “compact” state that was adopted by VSGs at the PM may facilitate incorporation of newly synthesized VSGs, whilst the previous VSG is being removed, ensuring that none of the non-variable antigens are exposed [37] (Figure 3). Similarly, the more “relaxed” state may be achieved after the antigenic switching program is completed [37]. The flexible states of VSGs are therefore potentially important for adaptation to a range of protein densities at the plasma membrane, of relevance during VSG switching as well as situations where additional membrane proteins are accommodated within a confined plasma membrane area [26,37] (Figure 3). 

Following translation, VSGs follow a trafficking itinerary to mature and be delivered to the surface [40]. VSG biosynthesis and transport is vital, as demonstrated by in vitro experiments in which the knockdown of VSG leads to marked defects in cell cycle, cell division, and survival [41,42]. The nascent VSG polypeptide is translocated to the ER in a Sec61-dependent manner, but likely to be independent of the signal recognition particle (SRP) [43,44]. This seems possible for most GPI-anchor proteins in *T. brucei*, whereas the translocation of polytopic membrane proteins requires SRP [45]. In the ER lumen, VSGs undergo multiple modifications, including the cleavage of the N-terminal signal and *N*-glycosylation and *O*-glycosylation, all being central to maintaining VSG expression levels and possible epitope diversification [46,47]. Importantly, there is some evidence to suggest that VSGs might be synthesised in excess, and their relative abundance being regulated by an active ER associated degradation (ERAD), which is consistent with the idea of altered surface VSG copy number during coat switching [48]. However, further evidence challenged these results, but argued in favour of the presence of active ERAD operating in the ER, acting as a quality control (QC) system for newly synthesized proteins, including membrane proteins [49]. 

Upon the addition of the GPI anchor and interaction with folding and QC systems, VSG is transported from the ER to the Golgi complex and matured further by glycan maturation [50,51]. Interestingly, the AP-1 complex is essential for biosynthetic lysosomal trafficking from the Golgi complex, at least in the PCF, and suggests a stage-specific role of this complex in *T. brucei* [28]. The role of the AP-1 in BSF remains to be fully elucidated for a greater repertoire of surface proteins in BSF, and thus merits further investigation. The final steps in delivery of the VSG to the PM are mediated by Rab11-containing vesicles and are likely coordinated by the exocyst [11]. The overall process (from translation to transport to the PM) is completed extremely rapidly, and due to the large excess of VSG polypeptides in the PM when compared to other organelles in the endomembrane system, indicates a concentration gradient towards the PM [26,40]. Nevertheless, mechanisms by which newly synthesized VSGs are concentrated, sorted, and QC controlled in different compartments of the endomembrane system remains elusive. 

During the transition from the vertebrate host to the insect vector (specifically, the tsetse fly), a major surface remodelling takes place, leading to a complete change in the high abundance proteins that are exposed in the surface coat. In the insect, the surface is initially dominated by procyclin, encoded by a gene family for GPI-anchored proteins with a characteristic amino acid repeat sequence; either Glu-Pro repeats (EP) or Glu-Pro-Glu-Glu-thr repeats (GPEET) [38,52]. Unlike VSG, the antigenic variation potential of procyclin is limited, reflecting the simpler and/or more limited immune response against the parasite within the insect host [52,53,54]. Procyclins are expressed in an orderly fashion, rendering parasites that simultaneously express both EP- and GPEET-procyclins [52,53,54]. During the onset of the infection in the insect host, the parasite express high levels of EP-procyclin, which levels then decrease following the transition from the midgut lumen to the epithelium (~3 days post-infection), coinciding with an expansion in the parasite population [52,53,54]. This eventually leads to a dominance of GPEET-procyclin at the cell surface [52,53,54]. This apparent orderly expression of procyclins in the cell surface might reflect specific interactions with the insect host at different tissues (midgut vs. epithelium vs. salivary glands), although this remains to be explored in detail. As proposed for VSG, the procyclin coat is thought to shield the parasite against proteolytic activity in the tsetse midgut, which is perhaps due to the presence of a high abundance of sialic acid on both *N*-glycans and the GPI-anchor, as well as extensive protein phosphorylation [52,53,54,55]. However, procyclin-null trypanosomes are capable of infecting tsetse, albeit at lower fitness [52,53,54]. Thus, while procyclin clearly provides a critical an advantage to the parasite, the reason for their switching within the insect host remains elusive, especially during later developmental stages within the tsetse, such as the epimastigote, which dispense with procyclins completely and express distinct surface proteins. 

## 4. Invariant Surface Glycoproteins

The most abundant trans-membrane proteins at the plasma membrane are the invariant surface glycoproteins, or ISGs [56] (Figure 3). Structurally, ISGs are type I trans-membrane domain (TMD) proteins that display some predicted structural similarity to VSGs, with an extracellular hydrophilic N-terminus, followed by a single transmembrane α-helix and a short cytoplasmic C-terminus [56]. There are at least five groups of ISGs, but only two have been analysed in any detail and are known to be expressed on the surface, ISG65 and ISG75 [56]. Both are only detected in the mammalian stage, suggesting that their functions are restricted to potential interactions with host-derived factors, and efforts to express ISGs in insect stages indicate the presence of a QC mechanism to prevent this from taking place and which involves the rapid degradation of the ISG [56]. 

Although the function of ISGs remain elusive, ISG75 (Tb927.5.400) plays a central role in mediating suramin internalization [57,58] (Figure 3). Suramin, the longest-standing trypanocidal drug, is a highly negatively charged molecule that is used to treat the haemolymphatic stage of trypanosomiasis [59,60]. Given its physicochemical properties, suramin cannot diffuse through the plasma membrane, suggesting that an active uptake process is required [59,60]. The current model for suramin uptake involves endocytosis via binding to ISG75 [57,59,60], as well as a role for lysosomal delivery of additional proteins by AP-1 complex [58]. Several lysosomal components are required for suramin sensitivity, including p67 (Tb927.5.1830) and CatL (Tb927.6.960), and thought to actively participate in degradation of ISG75 and subsequent release of suramin into the lysosomal lumen [58].

ISG75 and ISG65 are modified by ubiquitylation at specific cytoplasmic lysine residues [61,62]. Addition of ubiquitin is important for the internalization of ISG75 and ISG65, promoting endosomal targeting and degradation [57]. Furthermore, two deubiquitylating (DUB) enzymes, TbUsp7 (Tb927.9.14470) and TbVdu1 (Tb927.11.12240), act upon ubiquitylated ISGs, mediating the removal of ubiquitin [57]. However, due to the loss of the adaptor complex AP-2, it is possible that the addition of ubiquitin acts as a sorting signal for trans-membrane proteins that are destined either for recycling or degradation. Moreover, given the apparent absence of selectivity at the PM, ubiquitylation may well act as a checkpoint for further trafficking through the endosomal compartments. Taken together, the evidence presented thus far suggests that suramin is rapidly taken up and accumulates inside bloodstream form of *T. brucei* as a consequence of the high endocytic rate of ISG75, whereby ISG75 likely acts as a carrier for suramin [57]. However, there is not yet clear evidence to support a physical interaction between suramin and ISG75, and the comparatively small shift in the suramin half maximal effective concentration (*EC*_50_) for ISG75 knockdown may suggest additional levels of complexity in uptake pathways. Further investigation into the structural aspects of the potential interaction between ISG75 and suramin are required.

## 5. The Flagellar Pocket and Contact with the Environment

The FP is a specialized invagination of the plasma membrane, acting as a focal point for the exchange of material between the intracellular space and the extracellular milieu [5]; the configuration of the FP and the cytoskeleton that subtends it is in fact a consequence of the evolutionary origins of eukaryotes and the Excavata lineage [5] (Figure 2). The FP membrane has a unique array of lipids and proteins, including several likely receptors, as well as several signalling components, including the phosphoinositide-3-kinase (PI3K) Vps34 (Tb927.11.15330) and the target of rapamycin (mTOR) signalling pathways [63,64]. Indeed, the association of several PI3K-dependent components important for endocytic flux are almost invariably located at the FP [5,64]. Moreover, a physical association between the FP with the Golgi apparatus and different components of the endosomal compartments ensures that the entire system operates with great efficiency and likely close coupling between different pathways [5,64]. 

Several membrane proteins display a marked localization to the FP and/or endomembrane compartments subtending it. One of these membrane proteins is the transferrin receptor (TfR), which is a heterodimeric complex comprising the GPI-anchored protein expression site associated gene (ESAG) 6 (ESAG6) (Tb927.7.3250) and the soluble protein ESAG7 (Tb927.7.3260) [65,66]. After uptake, the TfR complex with transferrin (Tf) and this complex is routed to lysosomes where transferrin is proteolytically degraded [67,68]. While the degradation products are released from the cells, iron remains cell-associated and the TfR complex is recycled to the membrane of the flagellar pocket [69,70]. 

The FP also plays a central role in immune evasion. For example, one of the mechanisms by which trypanosomes are killed by the vertebrate immune system involves the induction of trypanosome cell lysis mediated by at least two different trypanolytic lytic factor (TLF) complexes, TLF1 and TLF2 [6,71,72]. TLFs are found in association with apolipoprotein-L1 (ApoL1), which is a component of the high-density lipoprotein (HDL) particles in human serum, and are actively internalized by trypanosomes via the haptoglobin-hemoglobin receptor (TbHpHbR) (Tb927.6.440), localized to the FP [6,73,74]. TbHpHbR acts as a receptor for haptoglin-hemoglobin and it is involved in the uptake and incorporation of heme into intracellular hemoproteins [75]. Upon internalization, ApoL1 undergoes a series of conformational changes in response to variations in endosomal pH, leading to the formation of pores in several intracellular membranes [71,72,76]. The trypanolytic action of ApoL1 is counteracted by the serum resistance-associated (SRA) protein (Tb927.9.17380), a GPI-anchored protein that is structurally related to VSGs [74], which directly binds to ApoL1 in the endosomal system, preventing the formation of membrane pores [71,72,76]. Although the mechanisms by which this process operates are well documented, the factors associated with the human serum TLF, contained in complex with ApoL1, were poorly understood. A recent RNAi-mediated screening surveyed the entire *T. brucei* genome and identified an array of proteins that are involved in the trypanolytic activity of ApoL1 [76]. Interestingly, at least six putative ubiquitylating enzymes that is involved in remodeling of the surface proteome (including the Really Interesting New Gene (RING)-E3 ligases and DUB enzymes), as well as several vacuolar ATPases, the lysosome-associated membrane protein p67 [77,78], and the TbHpHbR were all identified as bona fide components of the ApoL1-mediated trypanosome killing pathway [76]. The ubiquitylation/deubiquitylation cycle is known to be central in mediating protein trafficking at the PM and through the endomembrane system [79,80]. Together, these findings underpin the importance of intracellular trafficking events and ubiquitin-mediated systems mediating surface remodelling, as central components in the resistance to human serum [76]. 

## 6. Complex Interactions between Drugs and Trafficking Revealed by Genetics

Several studies have surveyed the *T. brucei* genome to identify proteins that are involved in the transport and metabolism of chemotherapeutic agents, either currently used or in development for treating early- or late-stage of HAT [58,81,82]. These demonstrated a robust link between transport mechanisms at the FP and sensitivity to chemotherapy [58]. Components required to mediate sensitivity to drugs include surface proteins that are predominantly located in, or trafficked through the FP, and components of, or interacting with, proteins mediating active internalization via endocytosis and/or recycling [58]. At least two proteins, ISG75 and aquaglyceroporin (AQP) 2 (Tb927.10.14170), mediate drug internalization; suramin and pentamidine/melarsoprol, respectively [57,58,83] (Figure 3). However, the mode of entry is context-dependent and depends on distinct pathways. Several polytopic proteins are also involved in susceptibility to difluoromethylated ornithine compounds (eflornithine), aromatic diamidines (pentamidine), and melaminophenyl arsenicals (melarsoprol) [58,83,84,85,86]. These include the amino acid transporter 6 (AAT6) (Tb927.8.5450), the plasma membrane P-type H^+^-ATPase (HA1-3) (Tb927.10.12510), the AT1/P2 adenosine/adenine transporter (Tb927.5.286b), and AQP2 [58,83,84,85,86].

Eflornithine, which is an irreversible inhibitor for ornithine decarboxylase (ODC), is efficiently transported by AAT6 [84]. The single-copy gene encoding AAT6 is involved in transport of neutral amino acids under physiological conditions [86], and has been identified as a target transporter by both metabolomics and genome-wide RNAi [58,81,87], thereby indicating its role in eflornithine sensitivity. Indeed, AAT6 RNAi knockdown increased the *EC*_50_ of eflornithine by ~16-fold [87,88].

Similarly, pentamidine and melarsoprol uptake are mediated by a myriad of trans-membrane proteins, including P-type H^+^-ATPase (HA1-3), the AT1/P2 transporter, and AQP2 [58]. Knocking down P-type H^+^-ATPases decreases the susceptibility to pentamidine 8-fold [58], and the current hypothesis proposes that the P-type H^+^-ATPase acts as a proton symporter generating the motive force that is required for pentamidine uptake, whereas the AT1/P2 transporter is involved in melarsoprol uptake [58,85,89]. Apart from the AT1/P2 transporter, the only other surface protein involved in pentamidine/melarsoprol cross-resistance is AQP2 [58,83,90], indicating a central role for these membrane-spanning proteins in the uptake both compounds. AQPs are conserved proteins that facilitate transport of small solutes across the plasma membrane, including water, urea, and glycerol [91,92]. Their main function is associated with osmoregulation [91,92]. *T. brucei* possesses three AQPs, all with distinct transport specificities [91]. Although these proteins display >70% similarity in sequence, several features distinguish AQP2 from AQP1 (Tb927.6.1520) and AQP3 (Tb927.10.14160), including a non-canonical NSA/NPS and IVLL amino acid motif alongside the pore channel and an unusual (and relatively wider) aromatic/arginine (ar/R) constriction [84,91], potentially explaining a capacity to allow for the passage of larger solutes. Similarly, AQP2 localizes almost exclusively to the FP, whereas AQP1 and AQP3 locate to the flagellum and the plasma membrane, respectively [83,93,94].

Owing to its localization in the FP, AQP2 may be subject to continued rounds of endocytosis and recycling. Indeed, in mammalian kidney epithelial cells, AQP2 traffics between the plasma membrane and cytoplasmic vacuolar reservoirs [92], which mediate the phosphorylation and ubiquitylation of human AQP2 [95,96,97,98]. There is also compelling evidence to suggest that phosphorylation of other AQPs has important functional consequences in both yeasts (*Saccharomyces cerevisiae*) [99] and *Leishmania major* [100]. Although evolutionary distant, the combined observations in yeast, kidney cells, and *L. major* provide a mechanistic framework to resolve the trafficking itinerary of the members of the AQP family. Several enzymes that are involved in post-translational modifications are also mediators of the susceptibility to both pentamidine and melarsoprol, including several E3 ubiquitin ligases, such as the cullin-RING protein (Tb927.11.11430), referred to as cullin 1, and at least one hypothetical proteins containing a C-terminal Homologous to E6-AP Carboxyl Terminus (HECT)-domain (Tb09.160.0350), suggesting that this is also likely an E3 ligases [58]. Similarly, several serine/threonine-protein kinases, such as STE7 (Tb927.8.5950), STE (Tb927.10.1910), mitogen-activated protein kinase 11 (Tb927.10.12040), and the nuclear Dbf2-related kinase (Tb927.10.4940) were also identified [58].

Interestingly, the serine/threonine protein kinase STE7 was detected as a mediator of susceptibility to both pentamidine and melarsoprol, while different E3 ubiquitin ligases are identified as specific for either pentamidine (cullin 1) or melarsoprol (HECT-domain hypothetical protein Tb09.160.0350) [58]. This observation may suggest that, although phosphorylation is central for susceptibility to both compounds, mechanisms controlling ubiquitylation-mediated internalization of these drugs may be divergent, involving distinct E3 ligases. It remains unclear whether the intracellular trafficking itinerary mediated by modifications of AQP2 is vital for trypanocide internalization. Alternatively, continuous cycling of AQP2 through the endo/exocytic machinery may act as a quality control mechanism controlling stability and correct (homo)tetramer formation, thereby ensuring a correct balance of functional AQP2 complexes in the cell surface, which in turn could facilitate the transport of trypanocides, such as pentamidine and melarsoprol through the plasma membrane. Further work is required to clarify the mode of internalization and action of these compounds by trypanosomes.

Although surface and FP proteins provide a route for entry into the cell, reverse genetics also indicates that proteins of intracellular organelles, such as the lysosome and the mitochondria contribute to drug sensitivity. For instance, knockdown of the glycoprotein p67, which is an essential lysosomal protein, decreases suramin sensitivity comparable to knockdown of ISG75 [58]. On the other hand, several aromatic diamidines, isometamidium chloride, and diminazene aceturate, important veterinary trypanocides accumulate in the mitochondria in a process that is dependent on the membrane-spanning mitochondrial vacuolar-type H^+^-ATPase [101,102,103,104]. Similarly, several mitochondrial enzymes, such as flavin-dependent nitroreductase (Tb927.7.7230) and flavokinase (Tb09.211.3420) and components of the ubiquinone biosynthesis, are involved in the susceptibility to nifurtimox [58], suggesting that the mitochondrion is one of the main intracellular target sites for several trypanocidal compounds. However, more mechanistic details are required to understand precisely how the surface composition of these organelles impacts drug sensitivity in trypanosomes, or how these compounds traffic between and are effectively delivered to subcellular organelles. 

## 7. Can We Harness Endocytic Machinery for Therapy?

Is it possible to harness the endocytic capacity, trafficking, and sorting of the bloodstream form of *T. brucei* to improve treatment of HAT? Although current therapeutic approaches are proficient in killing these parasites, issues with systemic drug toxicity and the emergence of drug resistance are shortcomings that could be bypassed altogether by targeting trafficking pathways. Moreover, a plethora of drugs have been developed through target-based strategies, and that fail against intact cells, which is likely due to difficulty in crossing biological membranes. In this regard, the development of nanobodies (Nbs), small (~15 kDa) antibody fragments derived from camelid heavy chains, has recently proved to provide an unparalleled opportunity to test this concept [105,106].

Nbs possess several key features that make them attractive candidates for therapeutic applications; apart from small size, they can bind to their target with nanomolar affinity and are highly soluble [105]. More importantly, given their small size they can recognize epitopes that are otherwise inaccessible to Immunoglobulin (Ig) G (IgG) or M (IgM), and they can penetrate the blood-brain barrier, providing an opportunity to deliver compounds to parasites in the Central Nervous System (CNS) during late stage disease [105]. A recent report demonstrated that nanoparticles loaded with pentamidine and coated with the Nb-33, which specifically recognizes a conserved N-linked high mannose oligosaccharide present on some VSGs, reduced the *IC*_50_ by 7-fold in vitro, and cured infected mice with a ~10-fold lower dose than free pentamidine [107,108]. An improved formulation reduced by ~100-fold the curative dose required when compared to free pentamidine in a murine model [107,108]. In this scenario, the pentamidine-loaded nanoparticle coated with the anti-VSG Nb specifically targets invariable epitopes of the VSG coat and is further internalized by a clathrin-dependent mechanism [12,105,106,107]. Owing to the rapid surface proteome turnover, this approach leads to a fast delivery and accumulation of pentamidine in the intracellular milieu, in a process that bypasses the mechanism of internalization that is mediated by AQP2 altogether, thus potentially overcoming resistance associated with mutations of AQP2 [12,105,106,107]. Although promising, several aspects need to be considered carefully before these findings can be effectively translated into clinical trials, as well as the consideration of alternative Nb targets and means for production. Perhaps one of the most challenging of these aspects is the discovery of novel epitopes that are unique to the parasites, with scope to reduce deleterious side effects or inadequate targeting. A second challenge would be to understand the capacity of these nanobodies to effectively and efficiently deliver different compounds through the blood-brain barrier, which is especially relevant for the treatment of late stages of trypanosomiasis. Other factors, such as nanoparticle type and formulation, route of administration, capacity of the nanoparticle to release its content in intracellular acidic compartments, stability in blood, as well as potential undesired side effects and bioaccumulation of nanoparticles in different tissues, are parameters that will dictate the success of this type of approach for clinical applications. Such studies are currently in progress in our laboratory and elsewhere.

## 8. Conclusions

*Trypanosoma brucei* maintains a surface proteome, which is mainly composed of VSGs in the bloodstream form, for the active evasion of recognition by both innate and acquired immune effectors. A highly rapid endocytic capacity is fundamental for the internalization and recycling of surface proteins, which in turn removes immune effector complexes bound to the cell surface. At the very core of this process, the flagellar pocket plays the central role in maintaining a flux of molecules to and from the plasma membrane, and the clear participation of conserved and lineage-specific proteins is now well established. What remains to be understood is a fuller account of the proteins that are targeted through the endocytic system and an understanding of how transport is regulated at different stages of the endolysosomal pathway. The small size of the trypanosome proteome, together with a comparatively simple endolysosomal system (when compared to mammalian cells) and an expanding suite of genetic tools, offers a possibly unique opportunity to address these questions in any organism. 

A promising avenue is development of novel chemotherapeutic agents. Many existing trypanosome drugs interact with surface proteins promoting their active internalization; essentially any compound that is charged at physiological pH requires a mechanism for translocation across either the surface or endosomal membrane. Significantly, the very high endocytic flux clearly explains the participation of ISGs in suramin sensitivity and potentially of pentamidine and AQPs. A recent demonstration that pentamidine-loaded nanoparticles that are coated with a nanobody recognizing an epitope associated with VSG was more efficient in killing trypanosomes by several orders of magnitude when compared to pentamidine alone, and bypassed resistance that is conferred by mutation of the AQP2 locus. Similar strategies directed against additional surface molecules are in progress and show considerable promise and they illustrate how understanding mechanisms underlaying maintenance of the surface proteome can aid in the development and application of novel approaches to tackle parasitic disease.
